# Spectrophotometric and Spectrofluorimetric Methods for the Determination of Dothiepin Hydrochloride in its Pure and Dosage Forms using Eosin

**Published:** 2010-12

**Authors:** M. I. Walash, F. Belal, N. El-Enany, H. Elmansi

**Affiliations:** *Department of Analytical Chemistry, Faculty of Pharmacy, University of Mansoura, Mansoura, Egypt*

**Keywords:** Dothiepin hydrochloride (DOP), eosin, spectroflurimetry, spectrophotometry, dosage forms

## Abstract

Spectrophotometric and spectrofluorimetric methods were developed for the determination of dothiepin hydrochloride (DOP) in different dosage forms. The spectrophotometric method (Method I) is based on formation of a binary complex with eosin at 540 nm in acetate buffer of pH3.7. The absorbance-concentration plot is rectilinear over the range 1–10 μg/mL with LOD of 0.18 μg/mL and LOQ of 0.54 μg/mL. The spectroflurimetric method (Method II) is based on the quantitative quenching effect of Dothiepin on the native fluorescence of eosin at the same pH. The quenching of the fluorescence of eosin was measured at 543 nm after excitation at 304 nm. The fluorescence-concentration plot is rectilinear over the range 0.3–8 μg/ mL with LOD of 0.11 μg/mL and LOQ of 0.34 μg/mL. The proposed methods were successfully applied to the analysis of commercial tablets and capsules containing the drug. Statistical comparison of the results with those of the reference method revealed good agreement and proved that there were no significant differences in the accuracy and precision between the two methods respectively.

## INTRODUCTION

**Figure 1:**
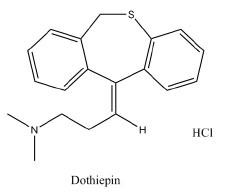
Structural formula of Dothiepin HCl (DOP).

Dothiepin hydrochloride (DOP) (dosulepin HCl; 3-(6H-di-benzo [b,e] thiepin-11-ylidene) propyldimethylamine hydrochloride; Fig. 1) (1) is a tricyclic antidepressant drug used widely to treat endogenous depression (2). The few analytical methods published for its determination include use of potentiometry (3), spectrophotometry (4-6), HPLC (7, 8) and GC-MS (9, 10).

Up till now, nothing has been reported concerning the spectrofluorimetric analysis of DOP. Spectrofluorimetric analysis is considered a sensitive tool, therefore, our target was to develop and validate simple and sensitive spectrophotometric and spectrofluorimetric methods for the determination of DOP especially in pharmaceutical preparations. Comparing to the other few reported methods, the two methods are sensitive, rapid, accurate and reproducible. The reported spectrophotometric methods require heating steps (4, 5) and this in turn affects reproducibility of the method. The other reported derivative method (6) is less sensitive than the proposed methods. The spectrophotometric method depends on measuring the product in the colorimetric region to avoid any interference from common tablet excipients. The reagent used is relatively inexpensive and its solution is stable for at least two weeks. The proposed methods are based on measuring the absorbance of the reaction product between eosin and DOP at 540 nm (Method I) or measuring the decrease in the fluorescence intensity of eosin upon addition of DOP at 543 nm after excitation at 304 nm (Method II). Both methods were successfully applied to the analysis of DOP in tablets and capsules.

## EXPERIMENTAL

### Apparatus

- The spectrophotometric measurements were established using Shimadzu UV-Visible 1601 recording Spectrophotometer (P/N 206-67001). Recording range, 0−1.0; wavelength 540 nm.
- The spectrofluorimetric measurements were made using Perkin Elmer LS 45 Luminescence Spectrometer equipped with 150 Watt Xenon arc lamp and quartz cell (1 cm).

### Materials

All materials and reagents were of analytical grade.

- Dothiepin hydrochloride was kindly supplied by El-Kahira, Pahrmaceutical and Chemical Industry Company Cairo, Egypt. The purity of the drug was found to be 99.23% according to the reference method (5);
- Prothiaden^®^ tablets each contain 75mg dothiepin HCl/tablet. Batch #73191;
- Prothiaden^®^ capsules each contain 25mg Dothiepin HCl/capsule. Batch #73222. Both are product of Kahira Pahrmaceutical and Chemical Industry Company Cairo. They were obtained from lacal pharmacy.

### Reagents

- Eosin (Riedel-De-Haen AG-D-3016 Seeize 1) 4 × 10^−3^ M aqueous solution for spectrophotometric method and 1.87 × 10^−5^ M aqueous solution for spectroflurimetric method. Both solutions were freshly prepared in distilled water and further diluted with the same solvent to the appropriate concentration.
- Acetate buffer solution (0.2 M) was prepared by mixing appropriate volumes of 0.2 M sodium acetate and 0.2 M acetic acid and adjusting the pH to 3.7 using pH Meter.
- Freshly distilled water was used.

### Preparation of stock and standard solutions

A stock solution of dothiepin HCl was prepared by dissolving 20.0 mg of dothiepin HCl in 100.0 mL of distilled water and was further diluted with the same solvent as appropriate. The standard solution was stable for 2 weeks when kept in the refrigerator.

### Construction of the Calibration Curves

**a) Spectrophotometric method (Method I).** Aliquots of DOP covering the working concentration range (1−10 μg/mL) were transferred into a series of 10 mL volumetric flasks and diluted to about 7 ml with distilled water. 1 mL of 4 × 10^−3^ M eosin solution was added to each flask, and the solutions were mixed well and diluted to the volume with acetate buffer (pH3.7). The absorbance value was measured at 540 nm against an appropriate blank prepared simultaneously. The measured absorbance was plotted vs the final concentration in μg mL^−1^ to get the calibration graph. Alternatively, the regression equation was derived.

**b) Spectrofluorimetric method (Method II).** The same procedure adopted for spectrophotometric method was followed except that, 2.8 mL of 1.87 × 10^−5^ M eosin was used and dilution of the standard solution to obtain the working concentration range of 0.3–8 μg/mL. The fluorescence intensity of the resulting solution was measured at 543 nm after excitation at 304 nm. The difference in the fluorescence intensity (ΔF) was plotted *vs* the final concentration of the drug (μg/mL) to get the calibration curve. Alternatively, the regression equation was derived.

### Applications

**Procedure for Capsules and tablets:** The contents of 10 capsules were emptied and mixed well. For tablets, twenty tablets were weighed and pulverized well. A weighed quantity of the powdered capsules or tablets equivalent to 20.0 mg of DOP was transferred into a small conical flask and extracted with 3 × 30 mL of distilled water. The extract was filtered into a 100 mL volumetric flask. The conical flask was washed with few mLs of water. The washings were passed into the same volumetric flask and completed to the volume with the same solvent. Aliquots covering the working concentration range were transferred into a series of 10 mL volumetric flasks. The procedure described under “Construction of calibration graph” was applied. The nominal content of the capsules or tablets was determined either from the previously plotted calibration graph or using the corresponding regression equation.

## RESULTS AND DISCUSSION

Eosin has been utilized for the determination of many pharmaceutical compounds of interest either through spectrophotometric measurment such as, fluoroquinolone antibacterials (11) gliclazide (12) ramipril and enalapril (13) or through spectrofluorimetric measurement such as, ramipril (14), fluphenazine and olanzapine (15) and some histamine H1-receptor antagonists (16).

The purposes of the present study were to develop simple and sensitive spectrophotometric and spectrofluorimetric methods for the determination of DOP in its pharmaceutical formulations without prior extraction procedures or using organic solvents.

**Figure 2:**
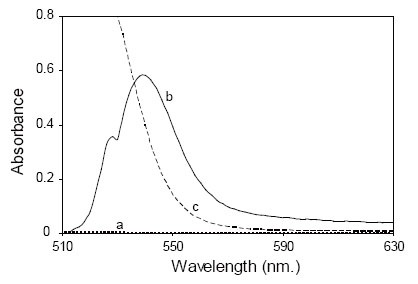
Absorption spectra of: a) dothiepin HCl only (6.0 μg/mL); b) Reaction product of dothiepin HCl (5.5 μg/mL) with (1.5 × 10^−4^ M) eosin at pH3.7; c) Blank eosin (1.5 × 10^−4^ M ) at pH3.7.

**Figure 3:**
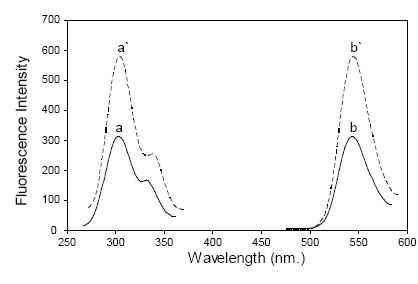
Excitation and emission spectra of: (a’, b’) Blank eosin (1.87 × 10^−5^ M) at pH3.7; (a, b) Reaction product of eosin (1.87 × 10^−5^ M) and DOP (8 μg/mL).

In the present study, DOP was found to form an ion pair red complex with eosin at pH3.7 with maximum absorbance at 540 nm (Fig. 2). The formed complex is mainly due to the electrostatic interaction between the studied drug and anionic functional group of eosin under acidic pH. The formed ion pair complex is not fluorescent; therefore, the decrease in the fluorescence of eosin upon the addition of the drug was the basis fot the spectrofluorimetric measurement at 543 nm after excitation at 304 nm (Fig. 3).

### Optimization of Experimental Conditions

The spectrofluorimetric and spectrophotometric properties of the product as well as the different experimental parameters affecting its development and stability were carefully studied and optimized. Such factors were changed individually while the others were kept constant. These factors include; the pH, type of buffer, volume of buffer, volume of eosin and time of reaction.

In spectrophotometric measurement, due to the slight solubility of the complex formed in aqueous solutions, and because eosin solution is more concentrated than that used in spectrofluorimetric method, it was difficult for the produced color to be accurately and precisely measured. Previous reports to solve such problem including: extraction with organic solvent has been an approach (17, 18) or addition of non ionic surfactants, such as methyl cellulose and Tween 80 (12, 19).

Upon addition of non ionic surfactants to prevent complex precipitation, the reproducibility was found to be adversely affected. Therefore, the method described by El-Brashy *et al*. (11) was adopted. It is based on keeping the sample concentration at maximum dilution before adding the dye solution, and mixing well before the addition of acidic buffer. Accordingly, the complex stability was achieved and precipitate formation was avoided with good precision.

**Figure 4:**
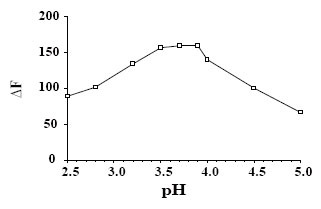
Effect of pH of 0.2 M acetate buffer on the decrease of the fluorescence intensity of eosin using 4 μg/mL of DOP.

**Effect of pH.** The pH is a critical factor in the complex formation, since it affects the ionization of eosin. The influence of pH of acetate buffer on the quenching of the fluorescence intensity of eosin was studied over the pH range 3–5. It was found that increasing pH values resulted in a subsequent increase in ΔF up to 3.5. This increase remains constant until pH4. After which a slight decrease in ΔF was achieved. Therefore, acetate buffer of pH3.7 was chosen as the optimum pH throughout the study (Fig. 4).

**Figure 5:**
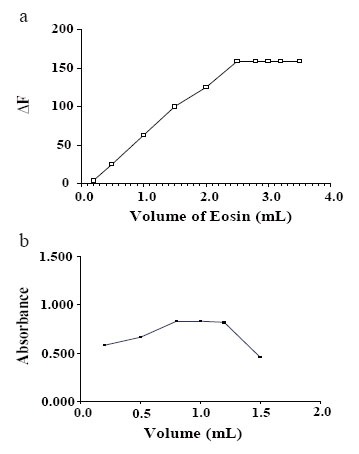
a) Effect of volume of eosin (1.87 × 10^−5^ M) on the decrease of the fluorescence intensity of eosin using 4 μg/mL of DOP. b) Effect of volume of eosin (1.5 × 10^−4^ M) on the absorbance of the reaction product of DOP (8 μg/mL).

**Effect of volume of eosin.** The optimum volume of the reagent was determined for the drug. For spectroflurimetric method; it was found that 2.8 mL of eosin (1.87 × 10^−5^ M) was sufficient to produce the maximum ΔF. For the spectrophotometric method 1 mL of eosin (4 × 10^−3^ M) was suitable to develop the maximum absorbance (Fig. 5).

**Effect of time of reaction and standing.** The effect of time on the quenching of the fluorescence intensity of eosin was also studied. It was found that the decrease in the fluorescence intensity of eosin was immediate upon addition of DOP and remained constant for more than 30 min. In spectrophotometric method; the intensity of the final color was stable for 48 h without precipitation of the complex.

### Analytical Performance

The absorbance-concentration plot was found to be linear over the range of 1−10 μg/mL. While, the difference in the fluorescence (ΔF) concentration plot was linear over the range of 0.3−8.0 μg mL^−1^. Linear regression analysis of the data are shown in Table 1.

Linear regression analysis of the data gave the following equations:

A=0.138+0.086C (r=0.9998)

ΔA=40.53+28.30C (r=0.9999)

where A is the absorbance in 1 cm cell, C is the concentration of the drug (μg/mL), ΔF is the native fluorescence of eosin solution (F°) - fluorescence of the reaction product (F), and r is the correlation coefficient.

The limit of quantitation (LOQ) was calculated according to ICH Q2 Recommendation (20) by establishing the lowest concentration that can be measured, below which the calibration graph is non linear and was found to be 0.54 and 0.34 μg/ mL for methods I and II respectively. LOQ was calculated from the following equation (20):

$$\text{LOQ}=10\;{\text{S}}_{\text{a}}/\text{slope}$$

The limit of detection (LOD) was also calculated according to ICH Q2 Recommendation (20) and was found to be 0.18 and 0.11 μg mL^−1^ for methods I and II respectively. LOD was calculated from the following equation (20):

$$\text{LOQ}=3.3\;{\text{S}}_{\text{a}}/\text{slope}$$

The proposed method were evaluated by calculating the accuracy as percent relative error and precision as percent standard deviation (RSD %) (Table 1).

**Table 1:** Performance data of the proposed method

Parameter	SpectrofluorimetricMethod	Spectrophotometric Method	Ref. method (12)

Concentration range (μg/mL)	0.3−8	1−10	50−250
LOD (μg/mL )	0.11	0.18	
LOQ (μg/mL)	0.34	0.54	
Correlation coefficient (r)	0.9999	0.9998	
Slope	28.30	0.086	
Intercept	40.53	0.138	
S_y/x_	1.42	6.14 × 10^−3^	
S_a_	0.96	4.67 × 10^−3^	
S_b_	0.22	7.89 × 10^−4^	
% Error	0.50	0.37	
% RSD	1.12	0.99	
Mean found (%)	100.17	99.58	101.30
± S.D.	1.12	0.99	0.38
Student’s t-value	0.044 (2.37)	0.05 (2.30)	
Variance ratio F-test	6.23 (6.59)	4.83 (5.41)	
Applications	tablets and capsules	tablets and capsules	

Values between parentheses are the tabulated t and F values respectively, at *p*=0.05 (26). S_y/x_, standard deviation of the residuals; S_a_, standard deviation of the intercept of regression line; S_b_, standard deviation of the slope of regression line; % Error, RSD%/√n.

### Validation of the method

The proposed methods were tested for linearity, specificity, accuracy and precision.

### Linearity

Under the described experimental conditions, the calibration graphs for the two methods were constructed by plotting the absorbance value in method I or difference in the fluorescence intensity (ΔF) in method II *vs* concentration in μg mL^−1^. The regression plots showed a linear dependence of ΔF and absorbance values on the drug concentrations over the range cited in Table 1. Regression equations, intercepts, slopes and correlation coefficients for the calibration data are presented in Table 1.

The validity of the method was evaluated by statistical evaluation of the regression lines regarding standard deviation of the residual (S_y/x_), standard deviation of the intercept (S_a_) and standard deviation of the slope (S_b_). The small values of the figures point out to the low scattering of the points around the calibration graphs and high precision (Table 1).

### Accuracy

Statistical analysis (21) of the results, obtained by the proposed and the reference methods for dothiepin HCl using Student’s t-test and variance ratio F-test, shows no significant difference between the performance of the two methods regarding the accuracy and precision, respectively (Table 1).

The reference method (5) is based on spectrophotometric measuring of the reaction product of DOP with 4-chloro-7-nitrobenzofuran (NBD-Cl) in the presence of 0.1 mol L^−1^ sodium bicarbonate at 470 nm after a fixed time 60 min at room temperature. It is tedious and time consuming method. Meanwhile, its reproducibility is poor and, in addition NBD-Cl as irritant reagent which preclude its use.

### Precision

**Repeatability.** The repeatability was performed through replicate analysis of two concentrations of the drug (2, 5 μg/mL) for Method I and (4, 10 μg/mL) for Method II in pure form on three successive times, and the results are shown in Table 2. The low values of standard deviations, % Error indicate high accuracy of the proposed methods, while low values of % RSD indicates high precision of the proposed methods (Table 2).

**Table 2:** Validation of the proposed method for the determination of dothiepin HCl in pure form

Sample concentration	% recovery (repeatability)	% recovery (Intermediate precision)

Method I		
2 μg/mL	100.8	100.45
	99.5	98.57
	99.0	100.34
X´	99.77	99.79
± SD	0.35	1.25
% RSD	0.35	1.25
% Error	0.20	0.72
5 μg/mL	100.34	99.43
	99.50	102.00
	99.98	101.67
X´	99.94	101.03
± SD	0.34	0.23
% RSD	0.34	0.23
% Error	0.20	0.13
Method II		
4 μg/mL	100.56	101.24
	98.67	101.55
	102.06	102.97
X´	100.43	101.92
± SD	2.40	0.99
% RSD	2.40	0.99
% Error	1.38	0.57
10 μg/mL	97.98	99.98
	98.57	100.93
	100.34	99.45
X´	98.96	100.12
± SD	1.25	1.05
% RSD	1.25	1.05
% Error	0.73	0.60

**Intermediate precision.** It was performed through repeated analysis of the drug in pure form, using the concentrations (2, 5 μg/mL) for Method I and (4, 10 μg/mL) for Method II for a period of three successive days. The low values of standard deviations, % Error indicate high accuracy of the proposed methods, while low values of % RSD indicates high precision of the proposed methods (Table 2).

### Robustness of the method

The robustness of the proposed methods was demonstrated by the constancy of the difference in the fluorescence intensity (ΔF) with the minor changes in the experimental parameters such as pH3.7 ± 0.2 and change in the volume of eosin, (1.87 × 10^−5^ M), using 2.8 ± 0.2 mL. These minor changes that may take place during the experimental operation didn’t greatly affect the decrease in the fluorescence intensity of eosin.

### Pharmaceutical Applications

The proposed method was applied to the determination of the studied drug in its dosage forms including tablets and capsules. Also the proposed method was tested for specificity and accuracy for tablets and capsules analysis (Table 3).

**Table 3:** Application of the proposed spectrofluorimetric method for the determination of dothiepin HCl in commercial tablets and capsules

Preparation	Spectrofluorimetric method		Ref. method (12)	
	Amount taken (μg/mL)	% Found	Amount taken (μg/mL)	% Found

Prothiaden tables (75.0 mg DOP HCl/Tablet)^a^	2.0	100.89	50.0	101.33
	4.0	101.55	100.0	101.56
	8.0	100.33	150.0	101.17
X± SD			100.92 ± 0.40	101.35 ± 0.20
Student’s t test			0.005	
Variance ratio F test			9.703	
Prothiaden capsule (25.0 mg DOP HCl/capsule)^b^	2.0	101.97	50.0	100.54
	4.0	101.12	100.0	101.24
	8.0	100.55	150.0	100.62
X± SD			101.21 ± 0.40	100.8 ± 0.44
Student’s t test			0.88	
Variance ratio F test			3.48	
Prothiaden tablets (75.0 mg DOP HCl/Tablet)	2.0	100.67	50.0	101.33
	5.0	100.54	100.0	101.56
	10.0	101.55	150.0	101.17
X± SD			100.92 ± 0.71	101.35 ± 0.20
Student’s t test			1.29	
Variance ratio F test			0.13	
Prothiaden capsule (25.0 mg DOP HCl/capsule)	2.0	99.98	50.0	100.54
	5.0	101.55	100.0	101.24
	10.0	101.20	150.0	100.62
X± SD			100.91 ± 0.25	100.8 ± 0.44
Student’\s t test			0.21	
Variance ratio F test			4.63	

The tabulated values of t and F are (2.78) and (19.00) respectively, at p=0.05 (26). Each result is the average of three separate determinations.

a Product of Kahira Pharm. & Chem.Ind. Co.;

b Product of Kahira Pharm. & Chem.Ind. Co.

### Specificity

The specificity of the method was investigated by observing any interference encountered from the common tablet and capsule excepients, such as talc, lactose, starch, avisil, gelatin, and magnesium stearate. These excepients didn't interfere with the proposed method.

### Accuracy

The results of the proposed method were compared with those obtained using the reference method (5). Statistical analysis (21) of the results obtained using Student’s t-test and variance ratio F-test revealed no significant difference between the performance of the two methods regarding the accuracy and precision, respectively (Table 2).

### Mechanism of the reaction

**Figure 6:**
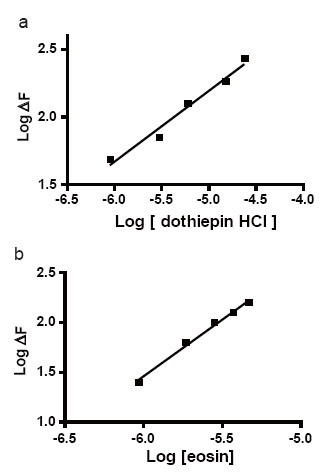
Stoichiometry of the reaction between drugs and eosin (1.87 × 10^−5^ M) adopting limiting logarithmic method. (A) Log [dothiepin] *vs* logΔF; (B) Log[eosin] vs logΔF.

**Figure 7:**
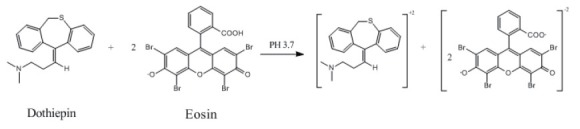
The proposal of the mechanism for the reaction between DOP and eosin.

The stoichiometry of the reaction between the studied drug and eosin was studied adopting the limiting logarithmic method (22). The decrease in the fluorescence intensity of the reaction product was alternatively measured in the presence of either eosin or DOP. Plots of log [DOP] vs log ΔF and log [eosin] vs log ΔF gave two straight lines, the values of the slopes were 0.52:1.13 for DOP: eosin respectively (Fig. 6). Hence, it is concluded that, the molar reactivity of the reaction is 1:2 drug: eosin. Based on the obtained molar ratio, A schematic proposal for the reaction pathway between the studied drug and eosin is shown in the following scheme (Fig. 7).

## CONCLUSION

The present study describes two sensitive methods for the determination of dothiepin without interference from common tablet excipients. From economic point of view, the proposed methods are simple, rapid and inexpensive. They also have wider linear range with good accuracy and precision beside the use of water as diluting solvent. The most important advantage of the methods is that the ion-pair formed is measured directly without need for pretreatment of the drug and extraction with organic solvent. Hence, it can be applied for the routine quality control of the studied drug in its dosage forms.
